# Usability Testing of an Internet-Based e-Counseling Platform for Adults With Chronic Heart Failure

**DOI:** 10.2196/humanfactors.4125

**Published:** 2015-05-08

**Authors:** Ada YM Payne, Jelena Surikova, Sam Liu, Heather Ross, Teodora Mechetiuc, Robert P Nolan

**Affiliations:** ^1^ Cardiac eHealth Peter Munk Cardiac Centre University Health Network Toronto, ON Canada; ^2^ Faculty of Medicine University of Toronto Toronto, ON Canada; ^3^ Ted Rogers Centre of Excellence in Heart Function Peter Munk Cardiac Centre University Health Network Toronto, ON Canada; ^4^ Department of Psychology McGill University Montreal, QC Canada

**Keywords:** chronic heart failure, self-care behaviors, e-counseling, usability assessment

## Abstract

**Background:**

Chronic heart failure (CHF) is a major cause of hospitalization and mortality. In order to maintain heart function and quality of life, patients with CHF need to follow recommended self-care guidelines (ie, eating a heart healthy diet, exercising regularly, taking medications as prescribed, monitoring their symptoms, and living a smoke-free life). Yet, adherence to self-care is poor. We have developed an Internet-based e-Counseling platform, Canadian e-Platform to Promote Behavioral Self-Management in Chronic Heart Failure (CHF-CePPORT), that aims to improve self-care adherence and quality of life in people with CHF. Before assessing the efficacy of this e-platform in a multisite, double-blind, randomized controlled trial, we evaluated the usability of the prototype website.

**Objective:**

The objective of the study was to assess the usability of the CHF-CePPORT e-Counseling platform in terms of navigation, content, and layout.

**Methods:**

CHF patients were purposively sampled from the Heart Function Clinic at the Peter Munk Cardiac Center, University Health Network, to participate in this study. We asked the consented participants to perform specific tasks on the website. These tasks included watching self-help videos and reviewing content as directed. Their interactions with the website were captured using the “think aloud” protocol. After completing the tasks, research personnel conducted a semi-structured interview with each participant to assess their experience with the website. Content analysis of the transcripts from the “think aloud” sessions and the interviews was conducted to identify themes related to navigation, content, and layout of the website. Descriptive statistics were used to summarize the satisfaction data.

**Results:**

A total of 7 men and women (ages 39-77) participated in 2 iterative rounds of testing. Overall, all participants were very satisfied with the content and layout of the website. They reported that the content was helpful to their management of CHF and that it reflected their experiences in coping with CHF. The layout was professional and friendly. The use of videos made the learning process entertaining. However, they experienced many navigation errors in the first round of testing. For example, some participants were not sure how to navigate across a series of Web pages. Based on the experiences that were reported in the first round, we made several changes to the navigation structure. This included using large navigation buttons to direct users to each section and providing tutorial videos to familiarize users with our website. We assessed whether these changes improved user navigation in the second round of testing. The major finding is that participants made fewer navigation errors and they did not identify any new problems.

**Conclusions:**

We found evidence to support the usability of our CHF-CePPORT e-Counseling platform. Our findings highlight the importance of a clear and easy-to-follow navigation structure on user experience.

## Introduction

### Chronic Heart Failure

Chronic heart failure (CHF) is a progressive clinical syndrome in which the heart is unable to pump oxygenated blood sufficiently to meet the metabolic demands of the body during exercise or at rest [1]. It is a major cause of hospitalization and mortality, and it is increasing in prevalence [2]. Prognosis is poor among patients who survive an index admission for CHF, with the 30-day hospital readmission rate at 35% [3]. The 5-year mortality rate is 45% for women and 60% for men [4]. Since there is no cure for CHF, quality of life is a clinically meaningful outcome for these patients [5].

Quality of life is a subjective, multidimensional construct that includes physical, social, and mental well-being [6,7]. CHF symptoms such as shortness of breath and fatigue [8] decrease functional capacity of patients, thereby impeding the pursuit of their life goals and reducing their quality of life [8,9]. Self-care behaviors (ie, recommended guidelines for heart healthy diet, regular exercise, medications, fluid and sodium intake restriction, symptom monitoring, and smoke-free living) are commonly prescribed to manage CHF and to improve quality of life [8]. However, long-term adherence to self-care has been low [10].

In an effort to improve quality of life, our research program has focused on developing telehealth and Internet-based counseling interventions (COHRT, I-START, and REACH) [11-17] that educate and motivate cardiac patients to adopt and maintain self-care behaviors. Our current trial, Canadian e-Platform to Promote Behavioral Self-Management in Chronic Heart Failure (CHF-CePPORT) [18], evaluates the efficacy of an e-Counseling platform in promoting self-care and quality of life among patients with CHF.

### Overview of Canadian e-Platform to Promote Behavioral Self-Management in Chronic Heart Failure e-Counseling Platform

This e-Counseling platform has been previously described [18]. In brief, it is an Internet-based preventive e-counseling protocol for patients with CHF. After logging onto the e-platform, users can access content that promotes: (1) explicit validation of the stage of “readiness” for behavior change, (2) active participation in the e-platform via self-guided navigation, (3) commitment to change by using “change talk” [19] to resolve ambivalence and reinforce motivation [20], (4) self-monitoring of behaviors identified by users as a priority for change, and (5) development of cognitive-behavioral skills to build and strengthen efficacy [21] as users embark on their behavior change. The counseling and educational content is reinforced through the use of multimedia, which we will describe below.

The content is organized into 28 e-sessions, delivered over a 12-month period. The e-platform proactively sends out 28 scheduled emails to inform users that new content is available. Each e-session consists of the four core features: (1) self-help video, which connects users with our CHF experts or other patients, and that reflects and validates the experiences of CHF patients; (2) educational content, which provides self-help information that supports users to best manage their condition; (3) interactive e-tools, which help to develop and strengthen self-care behaviors; and (4) e-trackers, which enable self-monitoring of behavior change. Users are self-guided to complete each e-session. A progress graph informs users of the proportion of the e-platform they have completed. They are encouraged to revisit any e-sessions they have previously accessed.

### The Need for Usability Assessment

Having a high quality, user-centered program would help maximize engagement and adherence to the e-Counseling platform [22]. To ensure that the e-platform is user-friendly, we conduced a usability assessment, which asked a sample of CHF patients to use the e-platform to perform predetermined tasks under a controlled condition while their experience was documented [23].

Other Internet-based self-management programs have conducted usability studies to help refine their prototype. For example, Stinson et al assessed the usability of an Internet-based, self-management program for adolescents with arthritis and their parents [24]. Voncken-Brewster et al assessed the usability of a Web-based behavioral self-management program for people with chronic obstructive pulmonary disease [25]. The above studies demonstrate that users can help to identify issues related to website design and functionalities that the program developers may have overlooked. Such findings can inform the refinement of the program and maximize its usability when it is fully deployed [22].

### Objective

This study examined the usability (navigation, content, layout, and satisfaction) of the CHF-CePPORT e-Counseling platform.

## Methods

### Study Design

We employed an iterative design [23,26], involving successive rounds of participants in this study. Feedback from the first round (hereafter known as Round 1) of participants was used to inform adjustments to the e-Counseling platform and then the revised version was tested in the second round (hereafter known as Round 2).

### Participants

We used inclusion criteria similar to the CHF-CePPORT trial to recruit participants: (1) male and female patients ≥ 18 years of age; (2) diagnosed with systolic CHF; and (3) fluent in English. We sampled individuals with varying degrees of experience with computers and the Internet to ensure that the e-Counseling platform was easy to use for both novice and advanced computer and Internet users.

Research assistants identified eligible participants from the Heart Function Clinic at the Peter Munk Cardiac Centre, University Health Network. We approached these individuals in person to introduce the study, solicit their consent to participate, and schedule an in-person study session with those who consented.

### Procedure

This study received ethics approval by the Research Ethics Board at the University Health Network. Each study visit was divided into two sections: (1) goal-oriented tasks and (2) feedback interview. The overall study visit took up to 1.5 hours.

Before each goal-oriented task, a research assistant read the instructions aloud. The participants were asked to “think-aloud” [27] as they completed each task. This protocol allowed us to directly capture the ongoing thought processes of the participants while using the program, as well as any difficulties they experienced [28]. Participants practiced the think-aloud protocol as they completed a set of sample tasks: they retrieved a nonpersonal standardized email from a sample email account and clicked on a hyperlink in that email. The link redirected them to the log-in page of the e-Counseling platform that we evaluated in this study. None of the participants reported any problems with the think-aloud protocol.

We asked participants to complete two goal-oriented tasks [23]. These tasks allowed us to identify any navigation problems in specific areas of the e-Counseling platform. The first task involved logging onto the website, watching an introductory video, and reading about the e-platform environment. This task assessed the ability of the users to navigate the e-Counseling platform (eg, using hyperlinks to move between pages) and to use an embedded video player. The second task involved completing an e-session that offered self-help tips and tools on active living with CHF. This sample e-session contained 4 core features: (1) a self-help video of exercise experts discussing self-help tips on living an active lifestyle and exercising regularly, (2) educational information that elaborates on the self-help tips mentioned in the above video, (3) an e-tool that helped users to set-up an exercise plan, and (4) a self-monitoring e-tracker of daily step counts. This task tested the ability of users to follow the self-guided session plan and to use the interactive e-tools and e-trackers without assistance.

The research assistants did not offer any help during the goal-oriented tasks, unless explicitly requested by individual participants [27]. This helped to minimize any disruptions to the spontaneous thoughts generated by the participants during task execution. We audiotaped all think-aloud sessions using a digital audio-recorder and then transcribed them verbatim. In addition, another research assistant acted as an observer and documented any problems that the participants may have encountered during the tasks [27]. Both think-aloud transcriptions and field notes were used during data analysis.

After completing the goal-oriented tasks, the research assistant interviewed each participant using a semi-structured interview guide, adopted from Stinson et al [24]. The interview questions focused on the website experience of the participants in three areas: navigation, content, and layout. The interview was also audiotaped on a digital audio-recorder and then transcribed verbatim for analysis. Finally, we asked participants to complete a demographics form and a satisfaction questionnaire. The items on the satisfaction questionnaire were based on the usability characteristics described by Nielsen [29].

### Data Analysis

The same protocol was used to analyze data from Rounds 1 and 2. We transcribed the audiotapes and verified the accuracy of the transcripts using a 2-person team: a research assistant transcribed the audiotape verbatim and another independently compared the transcript with the audiotape to check for accuracy.

We conducted a content analysis of the transcripts to identify issues in the three key areas of the e-Counseling platform: navigation, content, and layout. The authors (AP and JS) independently identified and categorized interview excerpts that described: (1) “successful navigation”, an incident when a participant was able to follow the website directions correctly or did not experience any problems using the website, for example, able to use a hyperlink to go to the next page, (2) “navigation errors”, an incident when a participant was unable to follow the directions provided on the website to complete an e-session or a feature of an e-session. For example, a user began an e-session on the wrong page, unsure of where to go after reviewing a page, or being unable to use a program feature even with instructions provided, and (3) positive and negative comments on various aspects of the website. Coding discrepancies were discussed and resolved between the two coders. Once the coding process was completed, the frequency count of each category was tallied. Pseudonyms were used when reporting any interview excerpts. Means (SD), and percentages were calculated for demographic and satisfaction data.

## Results

### Participant Description

There were seven individuals who participated in this study, with 4 participants in Round 1, and 3 in Round 2. The sample sizes were determined by data saturation [30]; that is, after the first 4 sessions, we did not identify additional unique issues raised by these participants, and therefore, we concluded Round 1. Similarly, after 3 sessions in Round 2, the participants did not experience the issues reported by those in Round 1, nor experienced any new issues. Thus, we concluded the study with 7 participants.

There were five men and 2 women who completed this study. The age of participants ranged from 39 to 77 years (mean 57, SD 14). There were 5/7 (71%) of them that were married and 6/7 (86%) that completed postsecondary education. There were 5/7 participants (71%) who self-identified as Caucasians, 1/7 (14%) as African American, and 1/7 (14%) as Chinese. Only 1/7 (14%) individual was currently employed.

All participants used computers and the Internet at home. There were 5/7 (71%) of them who were considered “intense users”, spending more than 5 hours per week on the Internet [31]. The individual who was working full-time also used computers and the Internet at work. On a scale from 1 (not at all comfortable) to 5 (very comfortable), Round 1 participants reported a mean comfort level of 4.5 (SD 0.6) with computers and a mean of 5.0 (SD 0.0) with the Internet, while Round 2 participants reported a mean comfort level of 3.3 (SD 1.2) with computers and a mean of 4.0 (SD 1.7) with the Internet. Table 1 provides more detailed descriptions of the 2 samples.

**Table 1:** Demographics and familiarity with computer and the Internet.

Demographics			Round 1 (n=4)		Round 2 (n=3)	
			n (%)	mean (SD)	n (%)	mean (SD)
**Age (years)**						
	30-45		1 (25)		1 (33)	
	46-60		1 (25)		1 (33)	
	>60		2 (50)		1 (33)	
**Gender**						
	Male		3 (75)		2 (67)	
	Female		1 (25)		1 (33)	
**Marital status**						
	Married/common-law		3 (75)		2 (67)	
	Single		1 (25)		1 (33)	
**Highest education level**						
	High school		1 (25)		0 (0)	
	College		1 (25)		2 (67)	
	Undergraduate degree		2 (50)		1 (33)	
**Current employment status**						
	Full-time		0 (0)		1 (33)	
	Disability/leave of absence		1 (25)		0 (0)	
	Unemployed		0 (0)		1 (33)	
	Retired		3 (75)		1 (33)	
**Ethnic background**						
	Caucasian		3 (75)		2 (67)	
	African-American		1 (25)		0 (0)	
	Chinese		0 (0)		1 (33)	
**Computer and Internet usage**						
	**Do you use the computer at home?**					
		Yes	4 (100)		3 (100)	
		No	0 (0)		0 (0)	
	**Do you use Internet at home?**					
		Yes	4 (100)		3 (100)	
		No	0 (0)		0 (0)	
	**Do you use the computer at work?**					
		Yes	0 (0)		1 (33)	
		Not applicable	4 (100)		2 (67)	
	**How many hours do you spend on the computer each week?**					
		≤ 5	0 (0)		2 (67)	
		> 5	4 (100)		1 (33)	
	**How many hours do you spend on the Internet each week?**					
		≤ 5	0 (0)		2 (67)	
		> 5	4 (100)		1 (33)	
	How comfortable are you with using the computer?^a^			4.5 (0.6)		3.3 (1.2)
	How comfortable are you with using the Internet?^a^			5.0 (0.0)		4.0 (1.7)

^a^ 1=not at all comfortable, 5=very comfortable

### Usability Findings

The study findings are organized into the following themes: overall satisfaction and general comments, navigation, content, and layout.

### Overall Satisfaction and General Comments

Table 2 summarizes the results of the satisfaction survey. Participants in both rounds were satisfied with the website, with all items having a mean score of 4 or above on a 5-point rating scale (1=disagree very much; 5=agree very much). There were minor differences on the M ratings between Rounds 1 and 2 participants on their mean item ratings.

**Table 2:** User satisfaction assessment.

	Round 1	Round 2
	mean (SD)^a^	mean (SD)^a^
I learned how to use this website quickly and easily	4.5 (0.6)	4.0 (0.0)
I can find the information I am looking for on this website with no problems	4.8 (0.5)	4.0 (0.0)
I can go through all the materials in an e-session with no problems	4.3 (0.1)	4.3 (0.6)
I am confident that I can remember how to get around this website on my own every time I log on	4.8 (0.5)	4.0 (1.0)
If I get lost on this website, I am confident that I can find my way again	4.8 (0.5)	4.7 (0.6)
I am satisfied with this website	4.8 (0.5)	5.0 (0.0)
I would use this website regularly to help me better manage my heart condition	4.8 (0.5)	4.7 (0.6)

^a^ Rating scale, 1=disagree very much; 5=agree very much

### Overall Feedback From Participants

Overall, all participants in both rounds of testing were very positive about the e-Counseling platform (see Table 3). They acknowledged the value of having self-care information accessible around the clock not only for new CHF patients, but also long-term patients. The time required to complete an e-session was deemed reasonable. They explored the website freely and believed that this website would be accessible to novice computer and Internet users. There were 2/7 (29%) participants that commented that the website felt like a personalized program. There were 3/7 (43%) participants that expressed that the website gave them hope that they can also live a heart healthy life, as one participant [P3, Round 2] said, “...this [website made] living healthy real to me. And achievable to me.”

**Table 3:** Content analysis, frequency counts of comments under each theme, and navigation issues.

Analyses		Round 1				Round 2			
		# C/I^a^	# of UCs^b^	# of P^c^	mean # C/I per P^d^	# C/I^a^	# of UCs^b^	# of P^c^	mean # C/I per P^d^
**Content**									
	Positive comments	62	27	4	15.5	39	15	3	13.0
	Negative comments	9	4	2	4.5	4	3	2	2.0
**Navigation**									
	Positive comments	16	16	4	4.0	30	4	3	10.0
	Negative comments	18	18	3	6.0	1	1	1	1.0
**Layout**									
	Positive comments	9	8	4	2.3	9	5	3	3.0
	Negative comments	6	3	2	3.0	1	1	1	1.0
**User navigation**									
	Correct navigation	70	14	4	17.5	67	10	3	22.3
	Navigation error	24	12	4	6.0	4	3	2	2.0

^a^ number of comments or incidents

^b^ number of unique comments

^c^ number of participants reported

^d^ mean number of comments or incidents per participants

### User Navigation

We identified a mean of 18 incidents of successful navigation per participant in Round 1 (see Table 3). These participants successfully logged in and out of the website, started the e-session, scrolled down the pages to review the content, navigated to the subsequent pages, and watched embedded videos. However, they also experienced a mean of 6 navigation errors per participant. There were 2/4 (50%) participants that wanted to make the video play on full-screen, but did not know how to do so. There were 3/4 participants (75%) that were uncertain about where to go next during the e-session. All 4 (100%) participants in Round 1 had some difficulties using the unfamiliar interactive features; for example, they did not know how to enter data into the e-tracker. Nevertheless, all the participants understood the purpose behind the interactive features and were willing to try them. They provided some suggestions to improve ease of use, especially for individuals who are less computer-savvy. These suggestions included providing more explicit directions on how to navigate the website.

**Figure 1:**
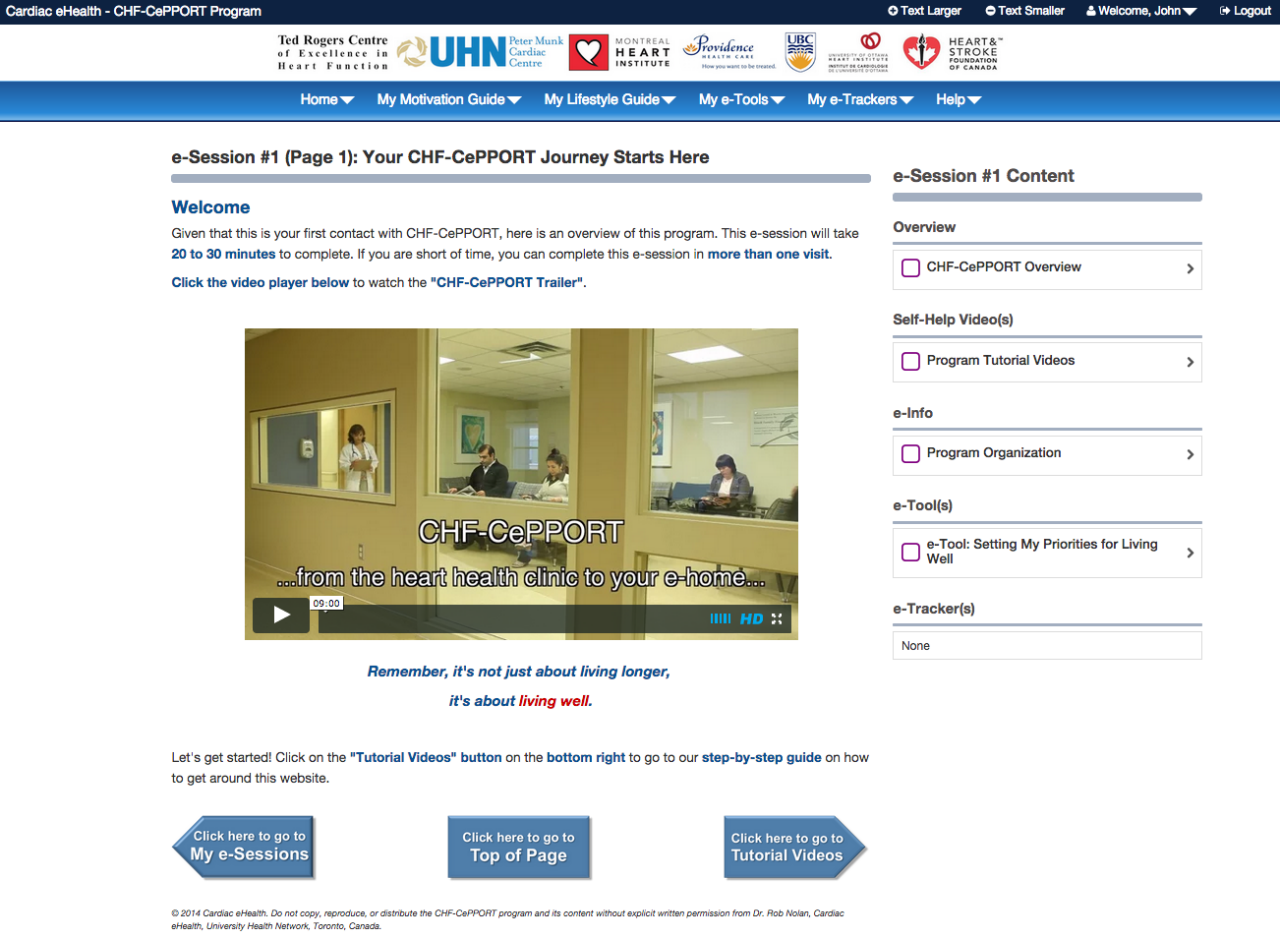
A screenshot of Canadian e-Platform to Promote Behavioral Self-Management in Chronic Heart Failure (CHF-CePPORT) e-Counseling platform after the second round of usability testing. Original image.

We modified the website based on these suggestions after Round 1, so that we could evaluate if the changes improved user navigation in Round 2. These changes included using large navigation buttons, instead of text hyperlinks, to take users to the appropriate content and to display clear notices to let users know when they completed an e-session (Figure 1 shows this). In addition, we added several tutorial videos that taught the participants how to use e-tools and e-trackers in order to improve users’ familiarity with these features.

Once these changes were made to the website, we observed improvements in navigation in the subsequent round. In Round 2, we identified a mean of 22 incidents of successful navigations and 2 navigation errors per person (see Table 3). There were 2/3 participants (67%) that experienced minor problems: one needed a reminder from the research assistant to scroll down the page for more content and the other did not enter a goal for step count in the e-tracker as instructed during the goal-oriented task. Ultimately, all participants in both rounds agreed that unlike other websites in which you can navigate freely, there is a learning curve to the self-guided structure of the CHF-CePPORT e-Counseling platform. However, the participants reported that they would have no problem navigating the website if they had a chance to use it once or twice at home. A participant [P2, Round 1] said, “...it will take a little time to get mastered and get on top of it, but I suppose any new website is like that and [this website is] a lot easier and more straightforward than any I’ve seen.” Since these last two minor issues did not significantly impede the successful completion of an e-session, we did not make further navigation-related modifications.

When we examined the comments related to navigation, we identified a mean of 4 positive comments and 6 negative comments per participant in Round 1 (see Table 3). In Round 2, participants made a mean of 10 positive comments per person and only 1 individual made a negative comment about navigation. Table 4 provides sample comments.

**Table 4:** Sample comments for each of the themes.

Theme and its definition	Example of a positive comment	Example of a negative comment
General comment:^a^ Comments made about the overall website	“...living with heart problem, I’m not by myself. There is something that could really help me.”[P3, Round 2] “I know that you’re doing this for a vast amount of people, but it really feels like this has been catered to me personally...”[P4, Round 1]	--- ^b^
Navigation: The ability for participants to independently move around the website, review the content, and use the e-tools and e-trackers as designed	“...for the first time, it’s figuring out, most of the time it told you that...the bottom right hand side of the screen to go to the next step and push the [button] and it took you to the next step...to me, that was important to know where the button was. Because sometimes they don’t tell ya and you’re looking, where is it?”[P1, Round 2]	“...hopefully I navigated the right way because it’s still not, from my perspective, completely intuitive as to once you come on to [the website], where you need to go.”[P1, Round]
Content: The material offered by the website, which can include self-help videos, didactic information, interactive e-tool, and self-monitoring e-trackers	“What I read most of was the exercise part and that was very helpful, very straight forward and the step counting and everything was very informative and it was very clear and quite complete and you have charts for schedules, which is a help. I found that very direct and straightforward.”[P2, Round 1] “...this is a guide, this for a self-help situation...what is being put here is to reinforce what I’ve already been told, what we’ve already been told [about self-care behaviors].”[P2, Round 2]	“...in this interview scenario, [the experts] are not facing you...if [they] were actually talking to the participant instead of each other that might be a better way of engaging someone?”[P3, Round 1]
Layout: The visual appearance of the website, including color, font size, images	“...it is a very attractive site. Yes, the pictures and it’s not just words and it’s well set up and arranged in charts so I think it’s very good...visually...”[P2, Round 1]	“...it might be a good feature maybe to have a font size where you can make it a little larger...”[P1, Round 2]

^a^ Individual participants were identified by a subject number and the testing round in which they participated.

^b^ All of the comments were deemed to be positive by both coders.

### Content

We identified a mean of 16 positive and 5 negative comments per participant in Round 1. In Round 2, we identified a mean of 13 positive and 2 negative comments per person in Round 2 (see Table 3).

Participants were very positive about the content of the e-Counseling platform, as indicated by the greater number of positive versus negative comments from both rounds of participants. All participants commented that the self-help materials were helpful, straightforward, approachable, and practical for cardiac patients and their families. The content covered a wide range of topics and reinforced the importance of adherence to self-care behaviors. Furthermore, the material addressed experiences faced by heart failure patients. A patient [P1, Round 2] said, “...[the content is] realistic to what you actually go through as a heart patient, the struggles you have going through...”

Participants also commented that videos and interactive tools helped to make the information easier to understand and the learning process more entertaining. There were 5/7 (71%) participants that thought the videos are of good quality. The experts in the videos offered useful information in an empathetic manner, which participants appreciated. There were 1/7 (14%) participant that commented that the daily step count e-tracker was a helpful tool to monitor self-care behavior change, while 3/7 (43%) participants appreciated having the progress graph to keep track of their progression through the CHF-CePPORT e-platform. To improve ease of use, 1/7 (14%) participant recommended the use of multiple-choice and check-box response options for some of the interactive tools. We plan to incorporate this suggestion in the next generation of the e-platform.

### Layout

We identified a mean of 2 positive and 3 negative comments per person about the layout in Round 1. We identified a mean of 3 positive comments per person regarding the website layout and only 1 negative comment in Round 2 (see Table 3).

Comments on the layout of the website were mostly positive in both rounds of testing. The participants remarked that the website had a clear and professional look. Its appearance was warm and friendly without being distracting to participants. A participant [P3, Round 2] remarked, “...the meat is the real thing. The real knowledge [on] how to take care of myself...instead of something flashing, or some colorful thing [to] distract me.” There were 2/7 (29%) participants that wondered if the font size was large enough for others with visual impairment. To address this, we subsequently added instructions, accessible at the top of every page, on how to adjust the font size through the Web browser.

## Discussion

### Principal Findings

The goals of this usability study were to gather feedback on the CHF-CePPORT e-Counseling platform and identify any navigation issues that may impede its usage. Although the findings highlighted some issues that our team did not anticipate, especially related to navigation, we also received many positive comments about the layout and content of the e-platform. This information was critical to the implementation of the e-Counseling platform in our multicenter, double-blind, randomized controlled trial [18]. By identifying and addressing usability challenges, we can ensure that these issues are unlikely to confound our trial findings.

Overall, the participants were very satisfied with the e-Counseling platform. Participants reported that it was easy to review the content and find the information for which they were looking. Most of our participants commented that the videos on our e-platform were of high quality and made them pay attention to the content. The learning process was entertaining and not time-consuming. This feedback was encouraging and supported our use of “edutainment” [32], for example, films that simulate real-life situations for patient education. This technique has been shown to improve accessibility and understanding of complex medical information (eg, medical tests and treatment options) among people of various levels of health literacy [33-35].

Our goal was to create an e-platform that is user-friendly for an older population because a significant portion of CHF patients are 65 years or older [36]. This segment of the population tends to be less computer literate than a younger age group, despite their growing engagement with the Internet [31]. Yet, they are also avid consumers of health information on the Web. Our Web design features incorporated recommendations made by the National Institute on Aging and the National Library of Medicine to ensure accessibility and ease of use [37]. These included using larger font size, white spaces around text, and a simple color scheme to improve readability. In this study, the participants were very positive about our layout and design. They commented that it was professional looking, while conveying warmth and friendliness. These participants did not have trouble reading the text, though a couple of them suggested offering a way to adjust the font size. Based on this feedback, we believe that the current e-platform design is suitable for older individuals with CHF.

A study goal was to identify and address any navigation issues. This was critical because such issues can impede users from accessing the clinical content, thus minimizing the effectiveness of the e-Counseling platform [38]. There were fewer navigation errors and negative comments made during Round 2. As a result, we believe that the changes we have made to the website after Round 1 have improved the accessibility of our website. This finding is even more encouraging because this improvement was observed from the participants in Round 2, who felt less comfortable with computers and the Internet than those in Round 1.

### Limitations

A few limitations should be noted. First, we relied on observations made by research personnel to document the interactions between participants and the e-platform. Using other methods such as video-capture of mouse clicks and screen display would provide more objective data on such interactions. However, the involvement of multiple independent data coders enhanced the rigor of our data analysis and interpretation process. Second, we tested the usability of the e-platform using a sample e-session. There are a total of 28 e-sessions in the CHF-CePPORT e-platform. We chose one of the e-sessions for testing to minimize respondent burden. Although this sample e-session included all the core features of the e-platform, there may be other usability issues in the remaining 27 e-sessions that we have not identified. Last, our sample of 7 participants may seem insufficient in assessing the usability of the e-platform. However, 80%-95% of usability problems can be identified using 5-9 individuals [39]. Moreover, we did not uncover additional issues from Round 2 participants. Thus, we are confident that the majority of usability problems on the e-Counseling platform have been identified and addressed.

### Conclusions

In this study, we found evidence to support the usability of the CHF-CePPORT e-Counseling platform. In addition to the content and layout, navigation proved to be a critical component for the design of our website. Internet-based self-management programs are becoming more common as a way to compliment medical therapies to manage complex diseases such as CHF. The CHF-CePPORT e-platform is consistent with our priority to design and implement an easy-to-follow navigational structure to facilitate user access.

## Abbreviations

CHF: chronic heart failure
CHF-CePPORT: Canadian e-Platform to Promote Behavioral Self-Management in Chronic Heart Failure
